# Extracellular Vesicles, Tunneling Nanotubes, and Cellular Interplay: Synergies and Missing Links

**DOI:** 10.3389/fmolb.2017.00050

**Published:** 2017-07-18

**Authors:** Muhammad Nawaz, Farah Fatima

**Affiliations:** ^1^Department of Pathology and Forensic Medicine, Ribeirao Preto Medical School, University of São Paulo São Paulo, Brazil; ^2^Department of Rheumatology and Inflammation Research, Sahlgrenska Academy, University of Gothenburg Gothenburg, Sweden

**Keywords:** extracellular vesicles, exosomes, tunneling nanotubes, heteroplasmy, neurodegenerative diseases, viral infections, tumor progression, therapies

## Abstract

The process of intercellular communication seems to have been a highly conserved evolutionary process. Higher eukaryotes use several means of intercellular communication to address both the changing physiological demands of the body and to fight against diseases. In recent years, there has been an increasing interest in understanding how cell-derived nanovesicles, known as extracellular vesicles (EVs), can function as normal paracrine mediators of intercellular communication, but can also elicit disease progression and may be used for innovative therapies. Over the last decade, a large body of evidence has accumulated to show that cells use cytoplasmic extensions comprising open-ended channels called tunneling nanotubes (TNTs) to connect cells at a long distance and facilitate the exchange of cytoplasmic material. TNTs are a different means of communication to classical gap junctions or cell fusions; since they are characterized by long distance bridging that transfers cytoplasmic organelles and intracellular vesicles between cells and represent the process of heteroplasmy. The role of EVs in cell communication is relatively well-understood, but how TNTs fit into this process is just emerging. The aim of this review is to describe the relationship between TNTs and EVs, and to discuss the synergies between these two crucial processes in the context of normal cellular cross-talk, physiological roles, modulation of immune responses, development of diseases, and their combinatory effects in tissue repair. At the present time this review appears to be the first summary of the implications of the overlapping roles of TNTs and EVs. We believe that a better appreciation of these parallel processes will improve our understanding on how these nanoscale conduits can be utilized as novel tools for targeted therapies.

## Introduction

Intercellular communication and the exchange of biological information between cells and organs is considered one of the sophisticated means of cellular coordination which modern eukaryotes have evolved to meet the needs of body physiology (Lai, 2004; Beckstead et al., 2006; Raft and Groves, 2015). Cells use different means of biological communication and signal transduction constituting direct physical contact between cells such as receptor-mediated interaction or cellular junctions between neighboring cells. Receptor-mediated cellular interactions are facilitated by certain transmembrane proteins and cell adhesion molecules such as integrins, tetraspanins, and cadherins (Hynes, 1992; Weber et al., 2011; Andreu and Yanez-Mo, 2014). The direct coupling of the cytoplasm of two cells through gap junctions (GJs) and concomitant transport of cytoplasmic material is also considered essential process in cellular cross-talk and is important for maintaining tissue homeostasis, development, and cellular differentiation (Schiller et al., 2001; Lim et al., 2011; Greco and Rameshwar, 2013; Munoz et al., 2013).

In the absence of direct physical contact, cells may convey biological messages in paracrine fashion through secreted factors such as cytokines, chemokines, and secreted growth factors (Sun et al., 2012; Tsuji and Kitamura, 2015). Therefore, the collateral effects of cells are not only reliant on direct cell-to-cell contact, but also to their transient paracrine actions through the release of a combination of trophic factors (Nawaz et al., 2016b). It is increasingly recognized that cells have evolved advanced forms of direct contact based communication and paracrine secreted factors. These include; (i) long range intercellular cytoplasmic bridges known as tunneling nanotubes (TNTs), and (ii) cell-derived nanovesicles known as extracellular vesicles (EVs). This review will illuminate biochemical, physiological, and pathological synergies between TNTs and EVs for their contribution in cellular communication and various pathological states.

## Biogenesis and physicochemical aspects of EVs and TNTs

Cells secrete heterogeneous population of nanovesicles that differ in their origins, modes of biogenesis, morphologies and sizes. International Society for Extracellular Vesicles (ISEV) has designated a generalized term extracellular vesicles (EVs) to represent heterogeneous populations of vesicles (Mateescu et al., 2017). Based on their biogenesis and release pathways (Nawaz et al., 2014), EV populations are classified into exosomes (40–120 nm) which originate from endocytic pathway, and microvesicles or ectosomes (100–1,000 nm) that shed directly from the plasma membrane. Recently, larger membrane derived vesicles known as large oncosomes (1–10 μm diameter) have also been reported as discrete class of EVs (Di Vizio et al., 2012; Minciacchi et al., 2015). Moreover, the apoptotic bodies are occasionally considered as a part of nanovesicles which are produced by indiscriminate apoptotic disintegration and are designated as apoptotic vesicles. Each subtype of EVs undergoes distinct biogenesis pathway where several factors participate in biosynthesis, sorting, and maturation of various populations of EVs and their secretion into extracellular milieu (for detailed mechanisms see Nawaz et al., 2014).

EVs are composed of lipid bilayer which primarily include sphingolipids, cholesterol and ceramide components and appear to have round shape or cup shaped morphology when observed under scanning electron microscopy. EVs are best characterized by the presence of integrins and tetraspanins on their surface such as CD9, CD63, CD81, and the cytoplasmic heat shock protein HSP70, and other proteins characteristic of EV components such as GAPDH, Tsg101 and Alix (Keerthikumar et al., 2016). These molecules usually serve as EV detection markers. Additionally, EVs surface may contain major histocompatibility complexes (MHC) such as MHC-I and MHC-II and adhesion molecules. Collectively these molecules define characteristic composition of EV populations. However, the biomolecular contents such as nucleic acids proteins, and lipids encapsulated within EVs differ greatly between individual EV subtypes or between EVs obtained from various sources depending on type and state of secreting cell.

TNTs are actin-based transient cytoplasmic extensions which are stretched between cells in the form of open ended nanotubular channels (50–200 nm) discovered by Rustom and colleagues (Rustom et al., 2004). Like EVs, TNTs also represent subtypes and heterogeneous morphological structures (Austefjord et al., 2014; Benard et al., 2015). However, biosynthesis of TNTs differs from EVs and is attributed to f-actin polymerization (Gungor-Ordueri et al., 2015; Osteikoetxea-Molnar et al., 2016). The regulatory pathways of TNT formation and endosomal trafficking are overlapped, both involving the components of exocyst complex which regulates vesicular transport from Golgi apparatus to the plasma membrane (Kimura et al., 2013, 2016; Schiller et al., 2013a; Martin-Urdiroz et al., 2016). M-sec, part of the exocyst complex interacts with Ras-related protein**-**A (RalA, small GTPase) and is required for TNT formation (Hase et al., 2009; Zhao and Guo, 2009). M-Sec in cooperation with RalA and the exocyst complex serves as key factor for the formation of functional TNTs and therefore M-Sec is considered TNT marker (Ohno et al., 2010).

Other studies demonstrate that formation of some TNTs might be actinomyosin-dependent (Gurke et al., 2008b; Bukoreshtliev et al., 2009). Perhaps not surprising, motor proteins are required for the generation of some forms of TNTs. For instance, myosin10 (Myo10) is required for TNT formation from filapodia, where the overexpression of Myo10 results in increased TNT formation and vesicle transfer between cells (Gousset et al., 2013). Elevation of Eps8 (an actin regulatory protein) inhibits the extension of filopodia in neurons and increases TNT formation as well as intercellular vesicle transfer (Delage et al., 2016). Several other mechanisms and molecular basis of TNT formation have been recently described elsewhere (Kimura et al., 2012; Ranzinger et al., 2014; Desir et al., 2016; Weng et al., 2016). A recent study has revealed the presence of actin-like filaments in a subpopulation of EVs, indicating that some EVs may possess an intrinsic capacity to move (so called motile EVs; Cvjetkovic et al., 2017). Altogether, these observations indicate that cells may use motor proteins as component of both TNTs and EVs for shipping their cargo.

## TNTs and EVs: synergies in cargo transfer and intercellular communication

**Figure 1:**
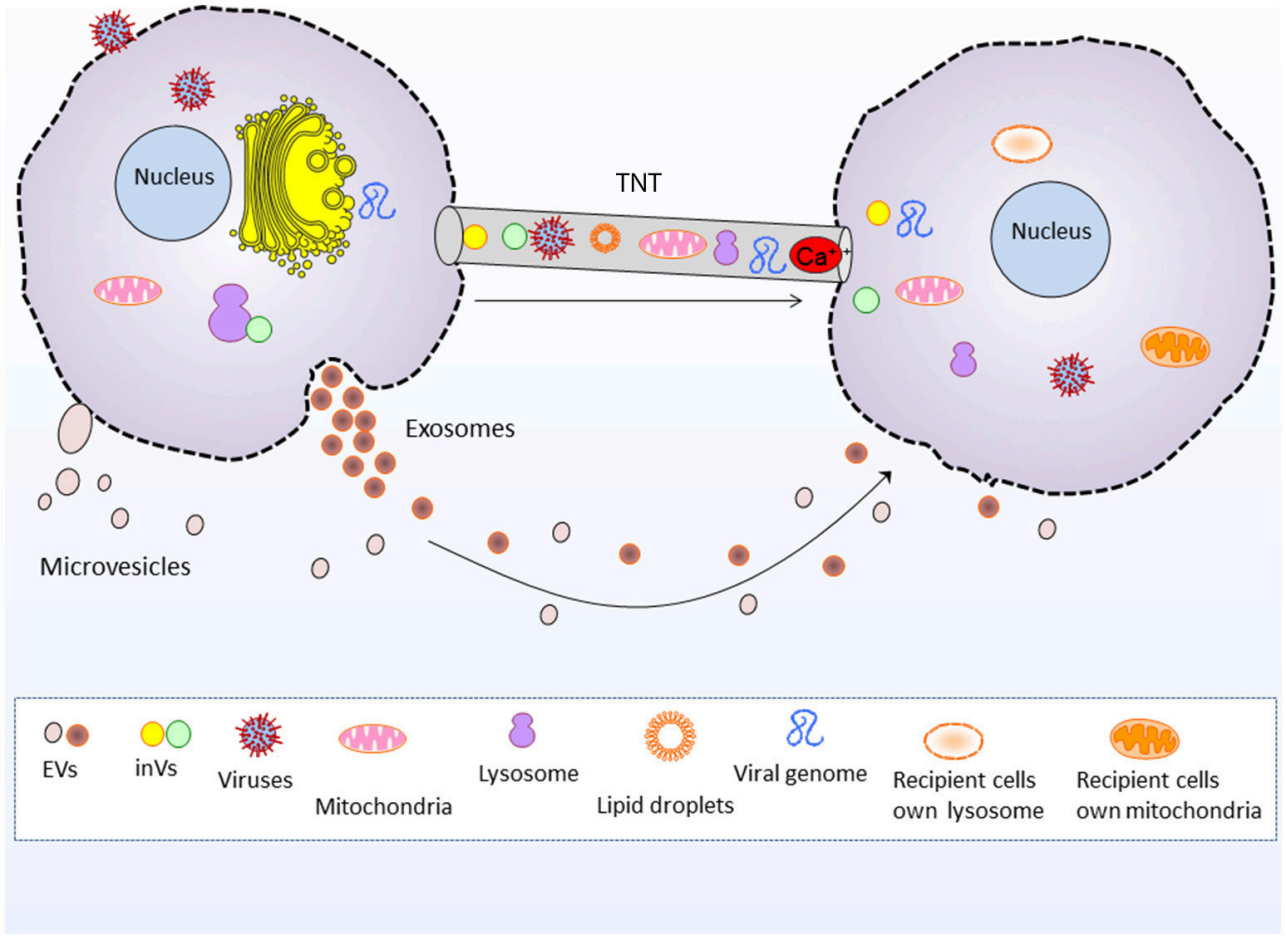
Tunneling nanotubes and extracellular vesicle mediated intercellular communication and cargo transfer. Tunneling nanotubes transport cellular organelles such as mitochondria and lysosomes, as well as viruses, viral genome, lipid droplets, intera-cellular vesicles and Ca^2+^ and electrical signals. Whereas, extracellular vesicles (exosomes and microvesicles) transport nucleic acids, proteins and lipids between cells. EVs, Extracellular vesicles, inVs, intra-cellular vesicles i.e., Golgi vesicles and lysosomal vesicles.

EVs are currently gaining intensive focus of interest in understanding their role in intercellular communication and dissemination of bioactive cargo. However, the communication assisted via TNTs is less well-known, and indeed it is an interesting area of research. Interestingly, EVs are implicated in transporting biomolecules bidirectionally (Nawaz et al., 2016b), same do the TNTs (Lou et al., 2012). Both EVs and TNTs could facilitate long range communication between cells. EVs transport biological material in paracrine fashion i.e., secreted from one cell and transported to other cell, whereas TNTs transport biological material through cytoplasmic bridges between cells located at long distance (Figure 1; Onfelt et al., 2005; Gurke et al., 2008a; Zani and Edelman, 2010; Zhang, 2011; Wang and Gerdes, 2012; Valente et al., 2015; Fykerud et al., 2016). Therefore, TNTs serve a unique way of rapid communication between long distance cells in the form of direct cellular conduits and thus, are considered distinct from other mediators of cell-cell communication or paracrine secreted factors (Frei et al., 2015).

EVs transport a repertoire of bioactive molecules such as lipids, proteins, and nucleic acids comprising genomic and mitochondrial DNA, mRNAs, miRNAs, and other ncRNAs (Fatima and Nawaz, 2015, 2017b). However, unlike EVs the TNTs are better known for shipping whole organelles by direct tubular connections between cells, such as mitochondria, lysosomes and Golgi vesicles (Rustom et al., 2002, 2004; Gerdes et al., 2007; Gurke et al., 2008b; Plotnikov et al., 2008; Wang and Gerdes, 2015; Han et al., 2016; Jackson et al., 2016; Torralba et al., 2016). The thicker subset of TNTs may range up to 0.7 microns (Onfelt et al., 2004; Benard et al., 2015), which is more favorable for the transport of larger organelles and lysosomal vesicles (Onfelt et al., 2006). TNTs also transport cytosolic Ca^2+^ and electrical signals to neighboring cells (Wang et al., 2010; Smith et al., 2011; Lock et al., 2016).

TNTs and EVs may exhibit the phenomenon of trogocytosis that is exchange of membrane fragments, for instance Fas-L and MHC molecules (Luchetti et al., 2012; Fatima and Nawaz, 2017a). In addition, TNTs may transport GM_1_/GM_3_ (gangliosides) containing vesicles, as well as intercellular exchange of B7-2 (CD86) molecules and MHC-II which represent novel pathways of intercellular communication and immunoregulation (Osteikoetxea-Molnar et al., 2016). Although, TNTs are characteristically known for organelle transfer however, like EVs they could also transport proteins and signaling factors (Gallagher and Benfey, 2005; Reichert et al., 2016; Zhang N. et al., 2016), lipid droplets (Astanina et al., 2015), nucleic acids such as miRNAs (Thayanithy et al., 2014b; Climent et al., 2015), and double-stranded small interfering RNA (Antanaviciute et al., 2014).

Increasing body of evidence clarifies that both TNTs and EVs are observed from diverse cell types, including immune, neuronal, stromal, cancer, and stem cells indicating their diverse roles in various physiological and pathological conditions. Organelle transport via TNTs generally represents the states of heteroplasmy, redox/metabolic homeostasis, and the concomitant pathological conditions (will be discussed in next sections). Similarly, molecular transport via EVs represents phenotypic and functional changes in recipient cells. Therefore, dissemination of various forms of cytoplasmic cargo mediated by TNTs and EVs exhibits multifaceted roles in human physiology and pathological states including immunomodulation, infectious diseases, neurodegenerative disorders, cancer progression, cellular homeostasis, and repair process that will be discussed in sections below.

## TNTs and EVs: roles in immunoregulation and inflammatory responses

Increasing body of evidence has demonstrated the contribution of EVs in immunomodulation and inflammatory responses both during normal physiology as well as pathological states (Zitvogel et al, 1998; Buzas et al., 2014; Robbins and Morelli, 2014; Nawaz et al., 2016a; Fatima and Nawaz, 2017a; Silva et al., 2017). However, the stimulatory roles of TNTs in cellular immunity are only recently beginning to be explored. TNTs have been shown to establish cytoplasmic bridges between variety of immune cells including human peripheral blood natural killer (NK) cells, EBV-transformed B-cells and the macrophages (Onfelt et al., 2004). Indeed, TNT formation in the context of immunity and inflammation such as antigen presentation (MHC complexes) has been widely reported in recent years (Chinnery et al., 2008; Schiller et al., 2013b; Seyed-Razavi et al., 2013; Campana et al., 2015; Osteikoetxea-Molnar et al., 2016). Arguably, such functional connectivity between immune cells may circumvent host defense against pathogens (Watkins and Salter, 2005; Zaccard et al., 2016).

Additionally, transfer of H-ras from B-cells to T-cells indicates that TNTs may activate ras signaling and other stimulatory effects in recipient cells suggesting their implications for immunity (Rainy et al., 2013). TNTs between primary cultures of patient derived human peritoneal mesothelial cells may present pathophysiological conditions associated with distribution of cholesterol levels and may stimulate inflammatory reactions (Ranzinger et al., 2011). Interestingly, senescence cells communicate via TNTs to regulate their immune surveillance by NK-cells and are thought to impact tumorigenesis and tissue aging (Biran et al., 2015). In this context, EVs have also been proposed to contribute in the processes of senescence and aging (Lehmann et al., 2008; Patel et al., 2016; Urbanelli et al., 2016; Eitan et al., 2017; Takahashi et al., 2017; Prattichizzo et al., in press). Although, many of the biological features are similar between EVs and TNTs (McCoy-Simandle et al., 2016), however it remains unclear whether EVs and TNTs act simultaneously and cooperatively during intercellular communication in the context of immune regulation. However, these are newly described modes of conveying immune responses being different from classical theories of cellular immunology.

## Resemblance in dissemination of disease associated patterns

### Neurodegenerative diseases

**Figure 2:**
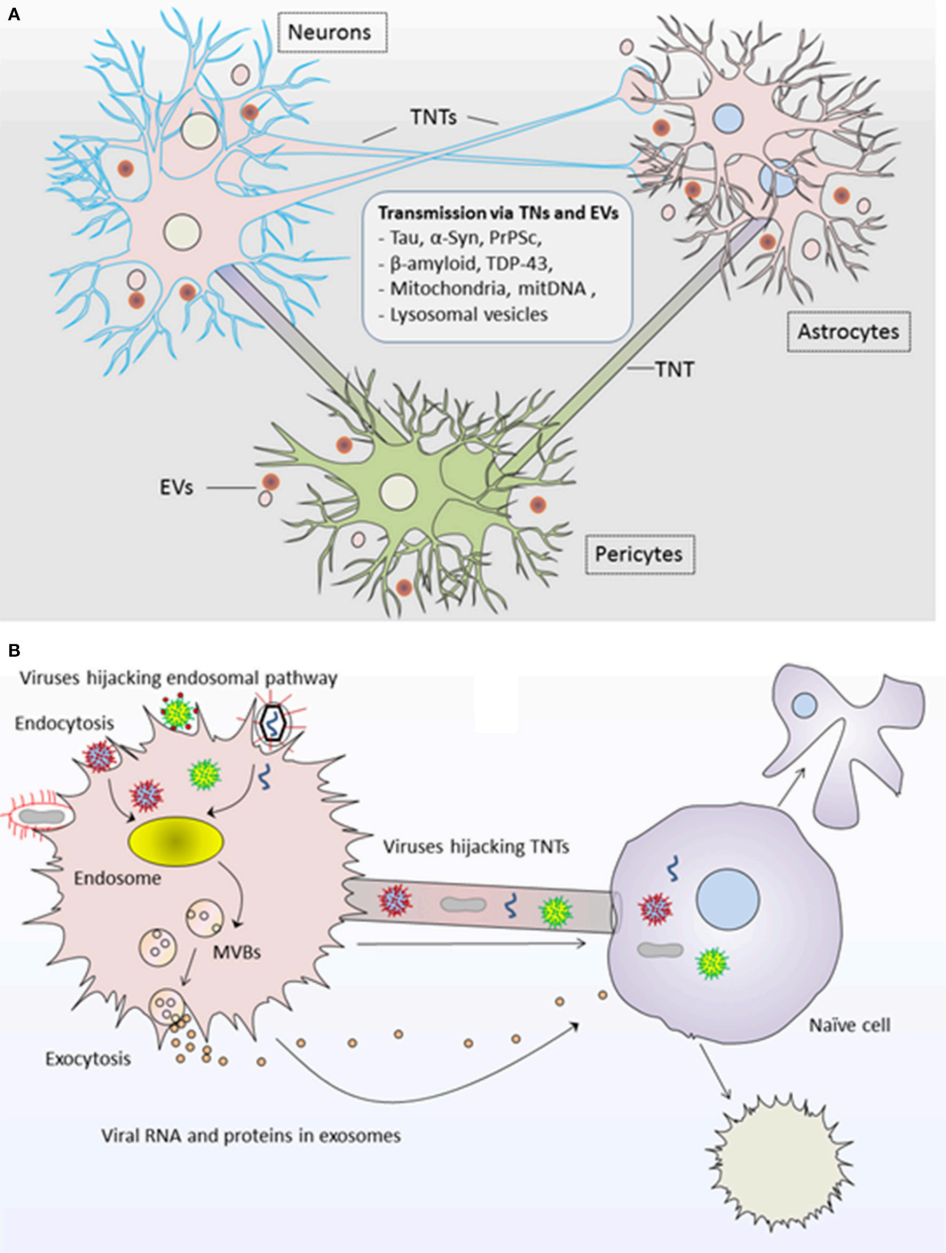
Roles of tunneling nanotubes and extracellular vesicles in pathogenesis of diseases. **(A)** TNTs and EVs transport neuropathogenic proteins and genetic content between neurons, astrocytes and pericytes and result into neurodegenerative pathology such as tauopathies and synucleinopathies including Alzheimer and Parkinson's disease. **(B)** Viruses highjack both TNTs and EVs for the propagation of viral infection. Viral RNA/proteins are incorporated into EVs via endosomal pathway and are transferred to unaffected naïve cells; whereas viruses may use direct transfer to naïve cells via TNTs. In both modes of propagation, the recipient cell may undergo cell death or transformed into infected cell pathology further spreading the infection to other cells. TNTs, Tunneling nanotubes; EVs, Extracellular vesicles.

Both TNTs and EVs have been implicated in the spread of misfolded protein aggregates between different cells of central nervous system (CNS). For instance, Tau and other prion-like proteins promote the formation of TNTs between neurons and thus their own intercellular transfer via TNTs which results in prion-like propagation of Tau assemblies and propagation of neurodegenerative pathology (Figure 2A; Zhu et al., 2015; Abounit et al., 2016b; Tardivel et al., 2016). Astrocytes use intercellular transport by TNTs and EVs for delivering mitochondria and neuropathogenic protein aggregates respectively and serve as mediators in the pathogenesis of Alzheimer disease (Engel, 2014). Moreover, EVs and TNT-like structure could supply the routes for the transfer of transactive response DNA-binding protein of 43 kDa (TDP-43) aggregates, whereas selective inhibition of their biosynthesis may interrupt the progression of TDP-43 proteinopathy (Ding et al., 2015). In fact, TDP-43 accumulation throughout the nervous system represents the development of neurodegenerative diseases such as amyotrophic lateral sclerosis and frontotemporal dementia (Ding et al., 2015). It has been proposed that intercellular dissemination of neuropathogenic proteins via TNTs could also cause damage to mitochondrial and/or mitochondrial DNA (mtDNA) in recipient cells and overall cellular degeneration (Agnati et al., 2010).

Additionally, fibrillar α-synuclein (α-syn) aggregates in lysosomal vesicles are transported between neurons via TNTs indicating the role of TNTs and lysosomes in the progression of synucleinopathies (Abounit et al., 2016a). TNT serve as conduits for α-syn transfer between non-neuronal cells during Parkinson's disease (Dieriks et al., 2017). Similarly, prion-infected astrocytes can disseminate prion (PrPSc) to neurons via TNTs and may contribute to disease progression (Victoria et al., 2016). In a way similar to viruses, the prions may highjack TNTs for spreading infectious agents such as PrPSc in neuronal cells (Gousset et al., 2009). Similar roles have been shown for EVs which transport α-synuclein, β-amyloid, and PrPSc and contribute in neurodegenerative diseases (Rajendran et al., 2006; Alvarez-Erviti et al., 2011; Bellingham et al., 2012; Guo et al., 2016; Loov et al., 2016; Vella et al., 2016).

### TNTs and EVs: novel routes of viral infection

Although, TNTs are characteristic of facilitating the exchange of organelles between cells, and pathogenic proteins from infected cells to naïve cells; however it remains unclear whether the viral genome is also transferred via TNTs and whether this route of transfer could result in replication of viral genome in the recipient cells. In this context, recent evidence show that influenza virus potentially exploits TNT networks for transferring viral proteins and the genome from infected to naïve cells (Kumar et al., 2017). Authors argue that influenza uses these networks for evading immune and antiviral defenses and provide an explanation for the propagation of influenza infections and vaccine failures. It has been shown that the icosahedral protein-rich membrane of virus could transform into a tubular structure (i.e., TNT), whereby internal diameter of the tube allows translocation of double-stranded DNA from one cell to the other (Peralta et al., 2013).

A protein encoded by the herpes simplex virus NV1066 named as enhanced green fluorescent protein (eGFP) was expressed in infected cells and transferred to uninfected recipient cells via TNTs. Particularly, the increased fluorescent activity was observed in recipient cells indicating the functional transmission of NV1066 virus expressed eGFP from affected to naïve cells (Ady et al., 2016). Moreover, following the viral thymidine kinase mediated activation of ganciclovir; TNTs could potentially mediate cell death as a form of direct cell-to-cell transfer. It is a unique form of long-range bystander effect underlying transmission of activated ganciclovir to nonvirus-infected cells. To this particular interest, authors argue that TNTs could be harnessed for the delivery of oncolytic viruses as well as viral thymidine kinase activated drugs in order to amplify the bystander effect between long distance cancer cells in a stroma-rich tumor microenvironments (Ady et al., 2016).

Cellular and molecular mechanisms that facilitate HIV infection in CNS could exploit the routes of intercellular communication via TNT for spreading the infection within CNS and may exhibit different forms of neuropathogenesis (Hazleton et al., 2010; Malik and Eugenin, 2016). It has been shown that HIV-infection of human macrophages induces the transient formation of short and long range TNTs, where distinct HIV vesicles are found to be localized in TNTs. This indicates that HIV hijacks TNTs communication to spread the pathogenesis of AIDS (Eugenin et al., 2009). Similarly, Retroviruses and Vaccinia virus could establish filopodial and TNT bridges between cells to spread the infection (Sherer and Mothes, 2008). TNTs have also been shown to facilitate HIV-1 transmission from activated T-cells to uninfected T-cells in a receptor-dependent manner (Sowinski et al., 2008, 2011). Transmission of HIV-1 via intercellular connections has been estimated far efficient than a cell-free process, perhaps in part explaining the persistent viral spread in the presence of neutralizing antibodies (Sowinski et al., 2008; Kumar et al., 2017). The coordination of exocyst complex and Nef protein (both are stimulators of TNT formation) is involved in polarized targeting for intercellular transfer of viruses and viral proteins (Mukerji et al., 2012; Hashimoto et al., 2016).

Like their role in TNTs, viruses could also highjack EVs for viral pathogenesis Figure 2B; Pegtel et al., 2010, 2011; Schwab et al., 2015; Nolte-'t Hoen et al., 2016; Sadeghipour and Mathias, 2017). In fact, viruses use EVs from their infected cells for delivering viral-RNA to uninfected cells, and this delivery is functional in recipient cells for the dissemination of viral infection (Pegtel et al., 2010). Interestingly, physical and chemical characteristics of biogenesis pathways of EVs resemble those of retroviruses, whereby viruses exploit the endosomal pathway for incorporating viral-RNA and viral-proteins into EVs being generated from virus-infected cells (Pegtel et al., 2011; Nolte-'t Hoen et al., 2016). Thus, viral factors from affected cells could be disseminated to uninfected cells via EVs and exhibit enhanced latent infection to surrounding cells (Schwab et al., 2015; Baglio et al., 2016).

### Resemblance in cancer progression

There is increased interest in understanding how EVs may facilitate tumor progression, metastasis, and development of resistance against therapies. However, the discovery of TNTs sheds light on novel mechanisms for networking between cancer cells and represents alternative way of transferring cellular contents that confer cancer progression and/or resistance to therapies. Several cancer cell types, in particular malignant mesothelioma cells including primary malignant cells from human patients have been extensively reported to exhibit efficient way of communication by TNTs and implicated in dissemination of oncogenic content (Lou et al., 2012; Ady et al., 2014; Antanaviciute et al., 2014; Desir et al., 2016). Interestingly, when mesothelioma cells were co-cultured with exogenous mesothelioma-derived EVs, the cancer cells exhibited accelerated rate of TNT formation indicating that EVs impact the TNT formation (Thayanithy et al., 2014a).

Xenograft model of breast cancer using eGFP-expressing mice showed that transferrin receptor is transferred from tumor cells to stromal cells *in-vivo* and this process is strongly correlated with an increased opposite transfer of eGFP from stromal to tumor cells. This suggests that TNTs mediate complex intercellular communication between stromal elements within tumor niche (Burtey et al., 2015). Tumor stromal cross-talk could also be explained from potential of TNTs in transferring oncogenic miRNAs via direct connections between cells (Thayanithy et al., 2014b). The similar mode of stromal cross-talk has been shown by EVs (Fatima and Nawaz, 2015; Webber et al., 2015; Choi et al., 2017). Although, EVs are implicated in the transfer of oncogenic miRNAs between cells; however TNT-mediated transfer seems to be distinct form of inter-cellular transfer.

TNTs between astrocytes and glioma cells facilitate transfer of oncogenic material and alter the proliferation potential of glioma cells (Zhang and Zhang, 2015). Interestingly, there has been shown a positive correlation between TNT formation and EV release in glioblastoma cells against cocaine in a dose dependent manner (Carone et al., 2015). This indicates the combined contribution of TNT and EVs in intercellular communication and glial-neuronal plasticity and may participate in the processes associated with cocaine addiction. Recently, it has been reported that TNTs could transfer microsized particles, which were produced by cancer cells in response to radio therapy (Ware et al., 2015). Importantly, cancer cells may use TNTs for developing resistance to therapies by transferring P-glycoprotein and mitochondria (Pasquier et al., 2012, 2013). Like TNTs, EVs have also been extensively demonstrated for their roles in multidrug resistance owing to transfer of biomolecules between cells that foster recipient cell properties to resist chemo/radiotherapies (Fatima and Nawaz, 2017b).

Chemotherapies to acute myeloid leukemia (AML) such as cytarabine and daunorubicin treatment has been shown to inhibit TNT formation (Omsland et al., 2017). Interestingly, daunorubicin was found to localize to lysosomes within TNTs formed between AML cells indicating a novel function of TNTs as drug transporting devices. Similarly, primary B-cell precursor acute lymphoblastic leukemia (BCP-ALL) cells communicate with primary mesenchymal stromal cells (MSCs) via TNTs which stimulates the secretion of prosurvival cytokines (Polak et al., 2015). This indicates that TNT signaling is important for the viability of patient-derived BCP-ALL cells. Moreover, TNT guided signaling induces stroma-mediated prednisolone resistance in B-cell precursor ALL cells. This is a novel communication mechanism by which ALL cells modulate their bone marrow microenvironment. The identification of TNT signaling in ALL-MSC communication gives insight into the pathobiology of ALL and opens new avenues to develop more effective therapies that interfere with the leukemic niche.

## TNTs and EVs: implications in regenerative/repair processes

The transfer of mitochondrial or mtDNA between mammalian cells including stem cells and mitochondria associated bioenergetics are increasingly being considered more dynamic process than previously thought (Spees et al., 2006; Rustom, 2016). MSCs are thought to transfer mitochondria to several different cell types including epithelial cells, endothelial cells, and cardiomyocytes (Plotnikov et al., 2008). In fact, such transfer may have positive effects on maintaining bioenergetics, enhancing cell survival, and ameliorating organ functions in conditions when mitochondria in resident cells are damaged and cells are facing oxidative stress. This includes the conditions of ischemic reperfusion, hypoxic or anaerobic conditions (Liu et al., 2014; Ham and Raju, in press), distressed cardiomyocytes and cardiomyopathy (Figeac et al., 2014; Yang et al., 2016; Zhang Y. et al., 2016), vascular injury (Vallabhaneni et al., 2012), intervertebral disc degeneration (Hu et al., 2015), corneal injury (Chinnery et al., 2008; Jiang et al., 2016), airway injury and allergic airway inflammation (Ahmad et al., 2014), acute lung injury (Islam et al., 2012), and acute respiratory disease (Jackson et al., 2016). As such, TNT-mediated intercellular communication and mitochondria transfer between stem cells and injured cells have multipotential ways to persevere cell and organ integrity. Interestingly, EV-mediated intercellular communication between stem cells and injured cells and concomitant dissemination of prosurvival growth factors, and angiogenic factors via EVs have been extensively implicated in tissue repair and ameliorating organ function (Nawaz et al., 2016b).

The contribution of TNTs in lysosomal transfer from endothelial progenitor cells to stressed endothelial cells has been attributed in reducing premature senescence and vasorelaxation (Yasuda et al., 2011). This healing effect is preserved by reconstituting lysosomal pool of stressed cells. Additionally, multisystemic lysosomal storage disease (termed cystinosis) could be repaired by TNT-mediated cross-correction (Naphade et al., 2015). Therefore, transport of cystinosin-bearing lysosomes into cystinosin-deficient cells via TNTs is a potential way of repairing lysosomal disorders (Gaide Chevronnay et al., 2016).

TNT-mediated communication between MSCs and renal tubular cells with extensive spontaneous intercellular exchange of cytoplasmic material contributes to renal physiology (Plotnikov et al., 2010; Domhan et al., 2011; de Cavanagh et al., 2014), as has been shown by MSC-derived EVs (Grange et al., 2014; Gu et al., 2016). Similarly, the regulation of TNTs between neural stem cells and brain microvascular endothelial cells could rescue brain function (Wang et al., 2016). TNTs facilitate peripheral nerve regeneration through the regulation of neural cell communications (Zhu et al., 2016), the same do EVs (Ching and Kingham, 2015; Lopez-Leal and Court, 2016). It has been reported that ribosome recruitment to axons occurs via lateral transfer from glial cells, a mechanism that could be part of development and a continuum of intercellular communication systems including TNTs and EVs (Twiss and Fainzilber, 2009).

## Concluding remarks and future directions

EVs are currently a focus of intensive interest in understanding their role in intercellular communication, dissemination of bioactive cargo, and their contribution in the progression of various diseases. Albeit, TNTs are comparatively less described nonetheless, they hold a great potential not only in studying cellular communication but also their multifaceted roles in disease progression and tissue repair. Given the fact that they transport organelles and regulate cellular bioenergetics; TNTs could be best exploited in treating organelle specific diseases in particular those associated with mitochondrial and lysosomal disorders.

Better understandings on the roles of nanotubes in tumor-stromal cross-talk could help to identify new selective targets for cancer therapeutics. Therefore, interfering with central routes of intercellular cross-talk via these membranous cellular tubes and EVs in separate or simultaneously could offer strong potential to explore novel strategies for directed therapy. If we develop mechanistic insights into the formation of TNTs and release of EVs, modes of cargo transfer, and their functional consequences; TNTs and EVs might one day be used as vectors of drug delivery against multiple diseases.

## Author contributions

Both authors participated in conceptualizing, writing, and critical review of the draft and agreed to final version before submission.

### Conflict of interest statement

The authors declare that the research was conducted in the absence of any commercial or financial relationships that could be construed as a potential conflict of interest.
